# Full recovery of near complete tear of anterior cruciate ligament without surgery: a case report

**DOI:** 10.1097/MD.0000000000019899

**Published:** 2020-05-01

**Authors:** Yoo Na Kim, Jungwon Baek, Young Hoon Kim, Jaewoong Hwang, Yu Ri Ko, Min soo Lee, Young chan Kim, Hue Jung Park

**Affiliations:** a Department of Anesthesiology and Pain Medicine, Seoul St. Mary's Hospital; b Department of Anesthesiology and Pain Medicine, Incheon St. Mary's Hospital, College of Medicine, The Catholic University of Korea, Seoul, Korea.

**Keywords:** anterior cruciate ligament, lateral collateral ligament, ligament tear, Polydeoxyribonuclotide

## Abstract

**Introduction:**

The anterior cruciate ligament (ACL) is the region where spraining or tearing is most prevalent when the knee is injured. Complete ACL ruptures have a much less favorable outcome without surgical intervention. Polydeoxyribonucleotide (PDRN) is a relatively safe substance widely used for regenerative therapy.

**Patient concerns:**

A 43-year-old female patient visited our clinic with Rt. knee pain after slipping, which she rated as 7/10 on a numeric rating scale.

**Diagnosis:**

She was diagnosed as having a near complete tear of the ACL at the femoral attachment, partial tear of the lateral collateral ligament.

**Interventions:**

Ultrasound-guided PDRN injections were carried out 5 times at intervals of about 2 weeks.

**Outcomes:**

At 3-month follow-up, the patient demonstrated an improvement in knee symptoms (numeric rating scale 0) and ROM without any complications. Even after 2 years and 5 months since the diagnosis, she has been doing her daily life well without any pain.

**Conclusion:**

This is the first report of successful PDRN injection for near complete tear of ACL and partial tear of lateral collateral ligament without surgery.

## 1 Introduction

The anterior cruciate ligament (ACL) is the region where spraining or tearing is most prevalent when the knee is injured. In America, ACL injury cases are estimated to occur in the range of 80,000 and 250,000 per year. ACL reconstruction surgeries are performed annually.^[1]^ Complete ACL ruptures have a much less favorable outcome without surgical intervention. ACL reconstructive surgery can be performed under a variety of anesthetic techniques including peripheral nerve blockade.^[2]^ Complications are rare in ACL surgery, but given the amount of ACL surgeries performed each year, the low rate still represent a significant amount of patient complications with potential for short- and long-term morbidity.^[3]^ Polydeoxyribonucleotide (PDRN) is derived from Oncorhynchus mykiss (Salmon trout) or O. keta (Chum Salmon), and is known as a substitute for glucocorticoids.^[4]^ It is a proprietary and registered drug that possesses tissue repairing, anti-ischemic, and anti-inflammatory activities.^[5]^ These therapeutic properties justify its use in regenerative medicine as well as in diabetic foot ulcers and cosmetics. Degenerative joint disease, or osteoarthritis, has been successfully treated in animals with PDRN; however, in humans there is still a lack of an effective. But the topical application of a regenerative gel containing PDRN has been shown to significantly improve pain and joint mobility with a clear amelioration of the clinical signs and radiological images^[6]^. The safety of PDRN has been established. Acute and chronic toxicity studies in mice and rats were undertaken to evaluate the effects of repeated systemic administration of PDRN. PDRN (8 mg/kg) showed no toxic effects in the brain, liver, lungs, skeletal muscle, and heart, and did not cause mortality^[7]^. In a trial investigating the effects of PDRN on the healing of chronic diabetic foot ulcers for up to 56 days, the safety and tolerability were found to be excellent^[8]^. We introduce a case of full recovery of near complete tear of the ALC and partial tear of the lateral collateral ligament (LCL) without surgery. The patient has provided informed consent for publication of the case

## 2 Case presentation

**Figure 1:**
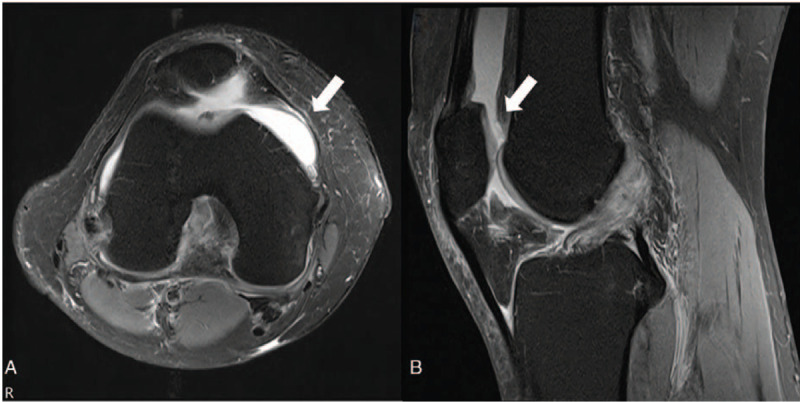
A) MRI knee Rt. (non-enhance sagittal view): immediately after injury. B) MRI knee Rt. (non-enhance axial view): joint fluid effusions.(arrow). MRI = magnetic resonance imaging.

**Figure 2:**
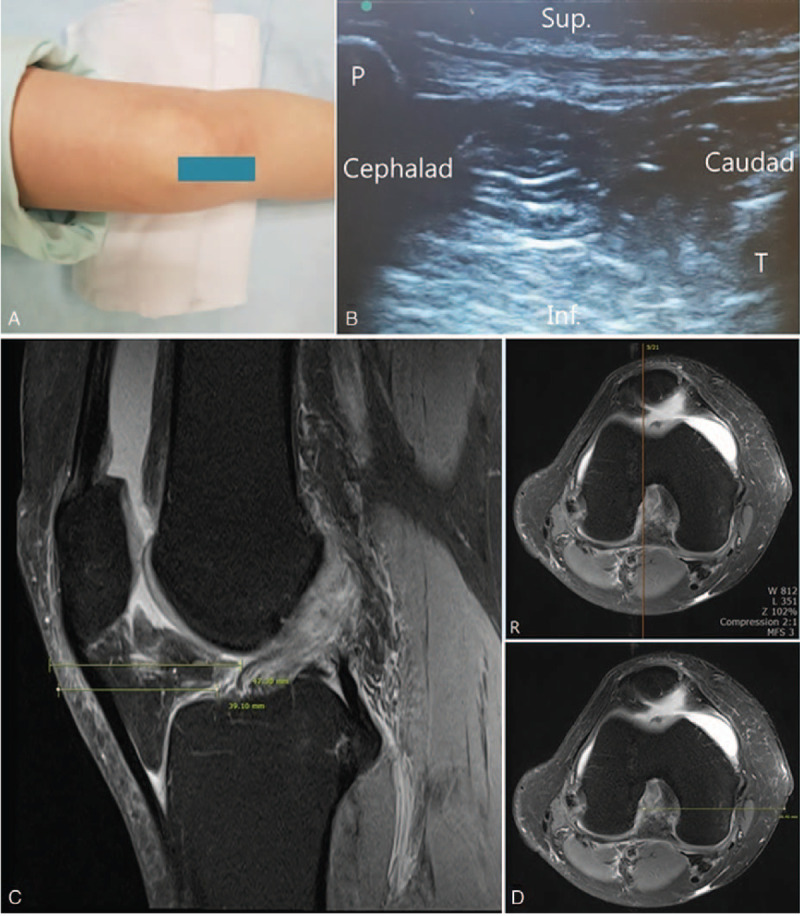
A) Ultrasound guided procedure of the knee: intraarticular injection. Longitudinal imaging over anterior knee.(square), B) Haziness of joint cavity, tibia (T), patella (P)., C) MRI knee Rt. (non-enhance axial view): Same level of the knee for measured a distance skin to target point., D) MRI knee Rt. (non-enhance sagittal view): Same level of the knee for measured a depth skin to target point. MRI = magnetic resonance imaging.

**Figure 3:**
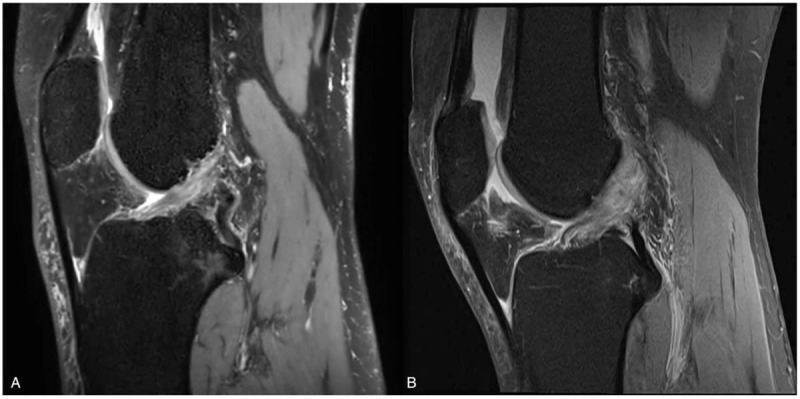
MRI knee Rt.(non-enhance sagittal view): comparison with follow up MRI (A) and immediately after injury MRI (B). MRI = magnetic resonance imaging.

A 43-year-old female patient visited our clinic with Rt. knee pain after slipping, which she rated as 7/10 on a numeric rating scale. She is a patient without a specific history. She could not walk on crutches without being in pain as well as limping and dragging her right leg. Rt. knee MRI findings showed near complete tear of the ACL at the femoral attachment, partial tear of the LCL, disruption of meniscopopliteal fascicle, and nonvisible popliteofibular ligament (Fig. 1). She also had joint effusion with loculated fluid in subpopliteal recess. First, the effusion was aspirated under ultrasound guidance. The hematogenous effusion was aspirated about 30cc. After joint effusion was aspirated and resolved, ultrasound-guided PDRN injections were carried out 5 times at intervals of about 2 weeks. The procedure was as follows: First, the triangular bony contours of the medial margins of the femur and tibia were identified, and the probe was placed over the medial knee joint in the longitudinal plane for injection. Using an out-of-plane approach, the needle trajectory is adjusted under real-time ultrasound guidance in order to enter the center of the knee joint through the triangular space. The needle does not appear in the ultrasound if it is well placed in the intra-articular space. The injection can begin once this has been achieved. With an intra-articular injection on the inferior side of the knee, patients usually have to pose with full flexion of the knee joint, because joint recess is easily palpated and exposed with full knee flexion. However, our patient could not move her knee because of severe pain. We were also concerned about an aggravation of the ligament tear. Thus, the procedure was guided with MRI. In order to determine the target point, we measured the depth of skin in MRI sagittal view (Fig. 2). We also measured the distance of the same level of MRI on axial view in order to inject at a more accurate site of the ACL. A mixture of 3cc of PDRN and 1cc of 1% lidocaine was injected into each of the ACL and LCL regions of the articular cavity by 2cc with a 25G 5 cm needle. The procedure was performed aseptically so as to avoid septic arthritis. Lee at al^[9]^ reported 2 cases of acute pseudoseptic local reactions after intra-articular hyaluronic acid injections in patients with knee osteoarthritis. The patient here was exhorted to wear braces for 12 weeks and to exercise regularly. At the 3-month follow-up, the patient demonstrated an improvement in the knee symptom (numeric rating scale 0) and ROM without any complications. Follow-up MRI showed partial reconstitution of the ACL and LCL (Fig. 3). It could clearly be seen that the tear improved through comparing the MRI images. Even after 2 years and five months since the diagnosis, she has been doing her daily life well without any pain. And there was no Adverse and unanticipated events. She is very satisfied with the results.

## 3 Discussion

Some ACL injuries occur in combination with damage to the meniscus, articular cartilage, or other ligaments. In addition, some patients may have bruises and effusions of the bone beneath the cartilage surface. The diagnosis of ACL rupture is often difficult to establish, particularly in recent injuries with acute hemarthrosis. In the case of suspected ACL injury, it is recommended to perform the pivot shift test, as it is highly specific and has a greater likelihood of discriminating and accurately diagnosing an ACL rupture. The Lachman test has great efficacy in ruling out a diagnosis of ACL rupture because of the lowest negative likelihood ratios.^[10]^ However, when MRI is available, all of these can be easily detected on a single MRI scan, and injury to the overlying articular cartilage can be indicated as well. Surgical treatment is usually advised in dealing with combined injuries.

However, deciding against surgery is a reasonable choice for certain patients. The nonsurgical management of isolated ACL tears is likely to be successful or may be applied in patients with the following conditions:

(1) those who with partial tears and no instability symptoms,
(2) those who do light manual work or live sedentary lifestyles,
(3) those whose growth plates are still open, and/or
(4) those who have severe underlying disease which increases the risk of any operation and general anesthesia, and who refuse operation.

Despite the fact that ACL injuries are usually treated surgically, the female patient in our case did not want to get any surgery.

Conversely, PDRN, whose action is derived from anti-inflammatory effects that promote wound healing by tissue regeneration, has no side effects.^[11,12]^ PDRN repairs and regenerates cellular damage by interacting with A2 purinergic receptor and stimulating the production of vascular endothelial growth factors.^[11]^ PDRN has recently found wide application throughout the medical field:^[13]^ not only in DM ulcer and cosmetics, but also in musculoskeletal areas. Even though PDRN does not seem to have an equivalent persistence effect compared to triamcinolone, considering the systemic side effects of steroids, particularly in patients with diabetes or metabolic syndrome, it appears that PDRN is worthwhile as an option for the treatment of hemiplegic shoulder pain.^[14]^ Mun et al^[13]^ reported the effectiveness of PDRN injection on pes anserinus bursitis. Lim et al^[11]^ reported the effectiveness of PDRN injection in posterior tibial tendon dysfunction patients undergoing ankle syndesmotic surgery. Kim et al^[15]^ demonstrated the effectiveness of PDRN injection in ischiofemoral impingement syndrome patients who were not indicated for surgery. Park et al^[16]^ applied PDRN injections into the carpal tunnel, and their patient demonstrated an improvement in carpal tunnel syndrome symptoms without any complications.

To the best of our knowledge, this is the first report of successful PDRN injection for near complete tear of the ACL and partial tear of the LCL without surgery. PDRN injection could be another option for patients with ACL injury who prefer nonsurgical treatment. We suggest that the safety and efficacy of PDRN injection should be researched further and administered more as a non-surgical treatment option.

## Author contributions

**Conceptualization:** Hue Jung Park ORCID (0000-0002-3775-1794).

**Data curation:** Jungwon Baek, Young Hoon Kim.

**Formal analysis:** Jaewoong Hwang, Yu Ri Ko.

**Project administration:** Min soo Lee, Young chan Kim.

**Supervision:** Hue Jung Park, Young Hoon Kim.

**Writing – original draft:** Yoo Na Kim.

**Writing – review and editing:** Hue Jung Park, Jungwon Baek, Jaewoong Hwang, Yu Ri Ko, Min soo Lee, Young chan Kim.
